# Emodin treatment is associated with enhanced resistance to *Aeromonas hydrophila* and correlates with gut microbiota–immune–metabolic modulation in Yellow River Carp: a multi−omics study

**DOI:** 10.3389/fimmu.2026.1881196

**Published:** 2026-07-15

**Authors:** Yuqing Liu, Shuaijie Sun, Juan Du, Bianzhi Liu, Guoxi Li, Guangqing Yu

**Affiliations:** 1 College of Animal Science and Technology, Henan Agricultural University, Zhengzhou, China; 2 Henan Academy of Fishery Sciences, Henan Academy of Agricultural Sciences, Zhengzhou, China; 3 Institute of Maternal and Child Health, Wuhan Children’s Hospital (Wuhan Maternal and Child Healthcare Hospital), Tongji Medical College, Huazhong University of Science and Technology, Wuhan, Hubei, China

**Keywords:** *Aeromonas hydrophila*, emodin, gut microbiota, immunology, Yellow River carp

## Abstract

**Introduction:**

*Aeromonas hydrophila* is a major pathogen of bacterial enteritis in aquaculture and a pathogen threatening the safety of fish products; sustainable alternatives to antibiotics are urgently needed. This study explored the potential effects of emodin in Yellow River carp (*Cyprinus carpio haematopterus*).

**Methods:**

Yellow River carp were used as a model, and multi-omics approaches (including 16S rRNA/ITS sequencing and untargeted metabolomics) were employed, along with evaluations of survival rate, hepatic and intestinal histopathology, quorum sensing-related virulence gene expression, and serum IgM and lysozyme activities.

**Results:**

Emodin treatment improved survival rate, alleviated pathological damage in the liver and intestine, downregulated quorum sensing-related virulence genes (*AhyR*, *LapA*, *AerA*), and enhanced serum IgM and lysozyme activities. 16S rRNA/ITS sequencing revealed that emodin increased the relative abundance of Ascomycota, Firmicutes, *Debaryomyces* and *Lactococcus*, while decreasing genera such as *Bosea* and *Legionella*. Untargeted metabolomics indicated marked changes in metabolic profiles. Correlation analysis revealed that the emodin-enriched microbiota was significantly associated with metabolites and the cytokines *il-1β* and *tnf-α*.

**Discussion:**

Emodin treatment was associated with enhanced anti-infection ability in Yellow River carp, concomitant with significant alterations in the intestinal microbiota, metabolism, and immune responses, underscoring its great potential as an environmentally friendly immunostimulant in aquaculture.

## 1 Introduction

The Yellow River carp (*Cyprinus carpio haematopterus*) is a unique and economically valuable fish endemic to the Yellow River basin, prized for its excellent meat quality and rich nutritional profile. As a widely consumed freshwater fish, its microbial safety has become a growing public health concern. However, the expansion of intensive farming and interbreeding with other strains have posed serious threats to this species, leading to reduced genetic diversity, weakened disease resistance, and degradation of its unique germplasm resources (1–4). Consequently, infectious diseases have emerged as a critical bottleneck limiting the sustainable and healthy cultivation of this species.

Among aquatic pathogens, *Aeromonas hydrophila* (*A. hydrophila*) is a primary causative agent of hemorrhagic septicemia and enteritis in freshwater fish (5–7). The pathogen also has the ability to produce and secrete multiple virulence factors (8). Infection by *A. hydrophila* induces clinical signs such as abdominal edema, liver and kidney damage, anal hemorrhage, and fin ulceration, often resulting in high mortality and substantial economic losses (9, 10). It is noteworthy that the immune responses related to disease resistance vary among genetic populations of different subspecies of common carp (11). As an important cultured subspecies in northern China, the Yellow River carp possesses a unique genetic background; however, its immune response characteristics upon *A. hydrophila* infection remain poorly understood.

For a long time, the extensive use of antibiotics has been the primary approach for controlling bacterial fish diseases; however, overuse has led to increased multidrug resistance in *A. hydrophila* and the issue of antibiotic residues (12). There is an urgent need for environmentally friendly alternative control strategies. Emodin, an anthraquinone derivative extracted from plants including rhubarb and *Polygonum cuspidatum*, has demonstrated potential in aquatic animals by promoting growth, exerting antioxidant and anti-inflammatory effects, and modulating the gut microbiota (13, 14). In common carp, dietary emodin supplementation may enhance intestinal immune defense by modulating the gut microbiota, thereby promoting the normal growth of the fish (15). However, most existing studies focus on phenotypic improvements and general measurement of immune parameters, without a systematic investigation into its underlying mechanisms. Particularly, the efficacy and molecular mechanisms of emodin in the Yellow River carp, a distinct subspecies, have not been comprehensively elucidated.

To address this knowledge gap, this study employs an integrated multi-omics approach to investigate the molecular mechanisms underlying emodin’s protection against *A. hydrophila* infection in Yellow River carp. This study not only provides a theoretical foundation for the application of emodin as a green feed additive in Yellow River carp aquaculture, but also provides a novel mechanistic perspective on herbal additives through the integrated lens of gut microbiota, metabolism, and immune response.

## 2 Materials and methods

### 2.1 Animal ethics statement

All experimental procedures involving animals were conducted in strict accordance with the guidelines of the Animal Care Committee of Henan Agricultural University (approval No. HNND2026010902). The animal experiments comply with the ARRIVE guidelines.

### 2.2 Yellow River carp, bacteria and emodin

Yellow River carp were supplied by Yanhui Wang from the Institute of Aquatic Sciences of Henan Province and acclimated to laboratory conditions for 14 d prior to experimentation. The fish were fed a commercial diet twice daily (07:00 and 17:00) at a rate of 1% of their body weight. The commercial feed was purchased from Tongwei Co.,Ltd. Additionally, water quality was managed daily, with at least one-third of the total volume changed. *The A. hydrophila* ‘type’ strain (ATCC 7966) is preserved by the College of Fisheries from the laboratory of Xianliang Zhao at Henan Normal University. The bacteria were inoculated into LB Broth and cultured at 28 °C for 24 h. The bacterial suspension was then centrifuged at 3000 rpm for 10 min. The supernatant was discarded, and the bacteria were washed three times with Phosphate Buffered Saline (PBS), then resuspended in sterile PBS to a final concentration of 1.0 × 10^7^ colony-forming units (CFU) per mL. Emodin (Purity ≥99.2%, HY-N0189R, MedChemExpress, China) was dissolved in dimethyl sulfoxide (DMSO) (Sigma Aldrich, purity ≥99.5%) at its maximum solubility of 5.41 mg/mL to prepare a stock solution. For oral gavage, the emodin stock solution was freshly diluted in sterile phosphate-buffered saline (PBS) to a working concentration, ensuring a final DMSO concentration of ≤ 0.1% (v/v).

### 2.3 Experimental design

The fish, with an average body weight of 10.17 ± 0.05 g, were randomly distributed into twelve 300-L plastic tanks, each containing 50 fish. The experimental design included four groups, each with three replicates: the control group (CON), the Emodin group (EM), the *A. hydrophila* group (AH), and the Emodin + *A. hydrophila* group (EMAH). The dosage of emodin supplementation was adjusted based on previous studies (16). Carp in the EM and EMAH groups were orally gavaged with emodin (50 mg/kg) once daily for 14 consecutive days. Fish in the CON and AH groups received an equivalent volume of the vehicle solution (sterile PBS containing the same final concentration of DMSO) via oral gavage on the same schedule, ensuring identical handling and vehicle exposure across all groups. Before the formal experiment began, the experimental fish were acclimatized for two weeks in 12 aquariums. During this acclimation period, the fish were fed and maintained under consistent conditions to ensure their health and stability. To eliminate tank effects during the formal experiment, the fish were randomly assigned to different treatment groups, and the aquariums were also randomly arranged and numbered. Each group contained three parallel replicates to allow for reliable statistical analysis. All experimental operations, including intragastric administration, injection, sample collection, and indicator measurement, were performed by trained operators who were completely unaware of the specific experimental group corresponding to each aquarium (intragastric administration and injection were each handled by dedicated personnel).

After the final gavage, fish in the AH and EMAH groups were intraperitoneally injected with *A. hydrophila* (1 × 10^8^ CFU/mL) at a dose of 1 μL per gram of body weight, based on the body weight of Yellow River car*p* (17). Fish in the CON and EM groups were injected with PBS instead. Mortality was recorded daily starting immediately after the injection for a period of 8 days. Survival curves were estimated using the Kaplan-Meier method, and the difference between groups was assessed by the log-rank (Mantel-Cox) test.

At 24 hours post−challenge (a time point determined by combining our preliminary experiment, in which clinical signs significantly worsened and mortality began, with reference (18)), fish were sampled. For biochemical assays and omics analysis, six biological replicates per group were obtained by randomly selecting two fish from each of the three replicate tanks (2 fish × 3 tanks = 6 samples per group); all omics samples were processed independently without pooling. They were first deeply anesthetized by immersion in 80 mg/L MS-222 (purity ≥98%, E10521, Sigma, USA) until reaching the surgical plane of anesthesia (total loss of equilibrium and reactivity, lying on the tank bottom with no effort to right themselves, but with continued opercular movement (19). Then, blood was collected from the caudal vein of 7 fish per tank (the other 6 fish were used for tissue collection as described below). Blood samples were centrifuged at 1250 × g for 15 min at 4 °C to obtain plasma for analysis of intestinal permeability and antioxidative capacity. All samples were stored at −80 °C until further analysis, except for the histological samples. After blood and tissue collection, the anesthetized fish were euthanized by immersion in an overdose of MS-222 (300 mg/L) for 20 min to ensure death, following the AVMA Guidelines for the Euthanasia of Animals (20).

### 2.4 Bacterial content detection

Intestinal, liver, and gill tissues were homogenized in 0.9% sterile saline at a 1:10 weight-to-volume ratio. The resulting homogenates were serially diluted, and 20 μL of each dilution was spread onto LB agar. Colony counts were performed after 12 h of incubation at 28 °C, with each sample tested in triplicate.

### 2.5 Total RNA extraction, cDNA synthesis and qRT-PCR

Total RNA was extracted from the liver and intestinal tissues of Yellow River carp using TRIzol reagent (TransGen Biotech, China) and reverse transcribed into cDNA according to the manufacturer’s instructions (Vazyme, China). qRT-PCR (Quantitative Real-time polymerase chain reaction) was performed using a Real-Time PCR Instrument (Thermo Fisher Scientific, USA), with the reaction setup based on the SYBR real-time PCR kit (Vazyme, China). The primers used in the assays are listed in **Supplementary Table 1**. Gene expression levels were quantified using the 2^−ΔΔCt^ method, with *β-actin* as the internal reference gene.

We adopted a specific absolute quantitative real-time PCR (qPCR) method targeting the AerA gene for the quantification of *A. hydrophila*. Conventional PCR amplification was performed using AerA-PCR primer sequences. The amplified products were verified by 1.0% agarose gel electrophoresis to confirm the expected fragment sizes, and then purified by gel extraction using the E.Z.N.A.™ Gel Extraction Kit. The purified PCR products were ligated into the pGEM-T Easy vector and subsequently transformed into competent *Escherichia coli* DH5α cells. The transformed cells were plated for blue-white screening, and positive clones were selected. Clones confirmed as positive by PCR were inoculated into LB liquid medium for expanded culture. A 1.5 mL aliquot of the bacterial culture was sent to a sequencing company for insert verification. The sequencing results were subjected to homology searches using BLAST on the NCBI website (http://www.ncbi.nih.gov/blast/). Plasmid DNA was extracted using the E.Z.N.A.^®^ Plasmid Mini Kit I Spin Kit, and its concentration was determined to calculate the gene copy number based on the plasmid concentration. A ten-fold serial dilution of the plasmid with known copy number was prepared as a standard template for subsequent quantitative qRT-PCR. The following formula was used to convert the cycle threshold (Ct) value to the copy number for the *A. hydrophila* gene: Ct = –3.0061 × log_10_(Copies) + 40.549, R2 = 0.9992 (21).

### 2.6 Histopathological analysis

24 h post-injection with *A. hydrophila*, liver and intestinal tissues from Yellow River carp were dissected, fixed overnight in 4% paraformaldehyde, and then processed through a graded ethanol series and xylene clarification. The samples were embedded in paraffin, sectioned into 4 μm thick slices using a microtome, stained with hematoxylin and eosin (H&E), and examined under a light microscope (Nikon Ti-E-A1R, Tokyo, Japan).

### 2.7 Biochemical analysis

Intestinal and liver tissue samples were homogenized in 0.9% sterile saline and then centrifuged at 3000 rpm for 20 minutes at 4 °C. The supernatant was collected, and oxidative indices, including Total superoxide dismutase (T-SOD), Total antioxidant capacity (T-AOC), Catalase (CAT), Malondialdehyde (MDA), Glutamic oxaloacetic transaminase (GOT), and Glutamate pyruvate transaminase (GPT), were measured using commercially available kits (Nanjing Jiancheng Bioengineering Institute, Nanjing, China) according to the manufacturer’s instructions. All experiments were performed in triplicate, and the results are presented as mean ± standard deviation (SD).

Frozen serum samples were thawed at 4 °C, and the levels of Complement Component 3 (C3), Complement Component 4 (C4), Immunoglobulin M (IgM), and lysozyme (LZM) in both serum and intestinal homogenates were determined using the Fish ELISA Kit (Shanghai Kqelisa Biotechnology Co.), following the manufacturer’s instructions.

### 2.8 Intestinal microbiome profiling

In this experiment, 16S rRNA gene and internal transcribed spacer (ITS) sequences amplicon sequencing was performed on samples of intestinal contents of Yellow River carp. Microbial DNA was extracted from intestinal contents using the MOBIOPowerFecal^®^ DNA Isolation Kit (Omega Biotek, Norcross, GA, USA), following the manufacturer’s protocol. The purity and concentration of the extracted DNA were assessed using a NanodropOne (Thermo Fisher Scientific, MA, USA). The V3-V4 region of the bacterial 16S rRNA gene was amplified using primers 319F (5’-CCTACGGGAGGCAGCAG-3’) and 806R (5’-GGACTACHVGGGGTWTCTAAT-3’). For fungal analysis, ITS2 was amplified using primers ITS3-F (5’-GCATCGATGAAGAACGCAGC-3’) and ITS4-R (5’-TCCTCCGCTTATTGATATATGC-3’). Sequencing was performed on the Illumina MiSeq PE300 platform (Meige Gene Technology Co., Ltd., Guangdong, China). Data processing and analysis were conducted using the OmicStudio tools available at http://cloud.magigene.com.

ASVs were obtained by DADA2 using 100% identity denoising to filter erroneous and chimeric sequences. ASVs were taxonomically annotated via qiime feature-classifier classify-sklearn against SILVA, RDP, Greengenes, UNITE and respective NR databases (default: SILVA 16S/18S, UNITE ITS, confidence = 0.8), with annotations assigned across seven taxonomic levels from kingdom to species. Venn analysis (VennDiagram, UpSetR R packages) was used to explore microbial diversity and interactions, while inter-group species comparisons were performed using Welch’s t-test and Wilcoxon rank-sum test. Alpha diversity indices (Chao1, Shannon, Simpson) were calculated with QIIME v1.9.1, and Kruskal-Wallis H test was used to determine inter-group significance. Beta diversity analysis was conducted via R vegan package combined with Jaccard and Bray–Curtis distance algorithms, and Adonis test (999 permutations) was applied to assess inter-group differences. LEfSe analysis was performed based on the normalized ASV table to identify differential species: Kruskal-Wallis (KW) sum-rank test was used for initial screening, followed by pairwise Wilcoxon rank-sum test, and LDA for dimensionality reduction (LDA score threshold = 2, regarded as group biomarkers). All results were FDR-corrected to eliminate false positives.

### 2.9 LC–MS conditions for metabolomics analysis

Intestinal content samples from Yellow River carp were prepared and deproteinized with methanol. Polar metabolites were then analyzed using LC-MS/MS, employing a UHPLC system (Vanquish, Thermo Fisher Scientific) coupled to a Waters ACQUITY UPLC BEH Amide column (2.1 mm × 50 mm, 1.7 μm) and an Orbitrap Exploris 120 mass spectrometer (Thermo). The mobile phase consisted of 25 mmol/L ammonium acetate and ammonia hydroxide (pH = 9.75) in water (A) and acetonitrile (B). The auto-sampler was set at 4 °C, and the injection volume was 2 μL. The Orbitrap Exploris 120 mass spectrometer operated in information-dependent acquisition (IDA) mode, controlled by Xcalibur software (Thermo), which continuously evaluated the full-scan MS spectrum. The ESI source conditions were as follows: sheath gas flow rate, 50 Arb; aux gas flow rate, 15 Arb; capillary temperature, 320 °C; full MS resolution, 60,000; MS/MS resolution, 15,000; collision energy, SNCE 20/30/40; and spray voltage, 3.8 kV (positive) or -3.4 kV (negative). Raw data were converted to mzXML format using ProteoWizard and processed with an in-house program developed in R, based on XCMS, for peak detection, extraction, alignment, and integration.

A total of 36,354 peaks were initially detected from 5 quality control (QC) samples and 32 experimental samples. To reduce systematic error, the following preprocessing steps were applied sequentially: (1) outlier filtering – peaks with a relative standard deviation (RSD, i.e., coefficient of variation) >30% were removed; (2) missing value filtering – only peaks with non−missing values in ≥50% of samples within at least one group or in ≥50% of all samples were retained; (3) missing value imputation – missing values were replaced by half of the minimum value; (4) normalization – peak areas were normalized using an internal standard. After these steps, 28,746 peaks were retained for further analysis. Pooled QC samples were prepared by mixing equal aliquots of all individual samples and were injected every 10 injections throughout the analytical run to monitor instrument stability and repeatability. Blank samples (extraction solvent only) were analyzed under the same conditions to assess potential contamination or carryover.

Metabolite identification was performed using the R package and BiotreeDB V3.0. The confidence of identification was classified according to the Metabolomics Standards Initiative (MSI) criteria: Level 1, identified by matching retention time, MS1 and MS2 spectra with authentic standards; Level 2, matched with public spectral databases (e.g., HMDB, MassBank) in MS1 and MS2; Level 3, matched with theoretical databases (e.g., Metlin) in MS1, MS2 and predicted RT; Level 4, unknown or MS1-only annotation. Based on acquired MS² spectra, 3,221 metabolites were finally annotated. Only these MS²-confirmed metabolites were used for subsequent differential expression and pathway enrichment analyses.

For multivariate statistical analysis, orthogonal projections to latent structures-discriminant analysis (OPLS-DA) was employed. Prior to modeling with SIMCA software (v16.0.2, Sartorius Stedim Data Analytics AB, Umeå, Sweden), data were log-transformed and scaled by unit variance (UV). The OPLS-DA model was constructed based on its first principal component; model quality was validated by 7-fold cross-validation, and model effectiveness was assessed using R²Y (interpretability of the categorical variable Y) and Q² (predictability). Model reliability was further verified by a permutation test (200 permutations), in which the order of the categorical variable Y was randomly shuffled multiple times to generate randomized Q² values. For univariate analysis, Student’s t-test was applied, and the false discovery rate (FDR) was controlled using the Benjamini-Hochberg method, with statistical significance set at FDR < 0.05. Differential metabolites were screened using the combined criteria: variable importance in the projection (VIP) > 1 from the OPLS-DA model and FDR-corrected P < 0.05. The relative abundances of differential metabolites were Z-score normalized and subjected to K-means clustering analysis. Based on KEGG pathway enrichment results, the Rich Factor was calculated as the ratio of differential metabolites annotated to a given pathway to the total number of metabolites in that pathway. Pathway enrichment P-values were derived from Fisher’s exact test and further corrected for multiple hypotheses using the FDR method. Graphs and heatmaps were generated using OmicStudio tools (https://www.omicstudio.cn/tool).

### 2.10 Statistical analysis

The experimental data are presented as mean ± standard deviation (SD). Statistical analysis was conducted using SPSS 26.0 (SPSS, Chicago, IL, USA). Shapiro-Wilk test and Levene’s test were used to evaluate data normality and homogeneity. For comparisons between two groups (e.g., bacterial load and virulence gene expression between AH and EMAH), an independent samples t-test was used. For comparisons involving all four groups (e.g., physiological, biochemical, and gene expression data), one-way ANOVA followed by Duncan’s multiple range test was performed. We chose Duncan’s test mainly based on the following considerations: (i) the number of treatment groups in this experiment was small (≤5), with equal sample sizes across groups; (ii) the data met the assumptions of normality and homogeneity of variances. Statistical significance was indicated at *P < 0.05.

The R packages “igraph” and “psych” were used to construct the microbial co-occurrence network, while Gephi software was employed for network visualization and beautification. For the analysis of relationships between differential metabolites and bacterial genera, the R packages ggplot2, ggpubr, and psych were utilized to calculate Spearman correlation coefficients. Relationships with correlation coefficients (cor) > 0.7 or < -0.7 were screened to construct the key bacterial genus-metabolite network, and the corresponding network diagram was drawn using Cytoscape. Additionally, Spearman correlation heatmaps between immune proteins, inflammatory markers, and bacterial genus parameters were calculated, and visualized using the OmicStudio tool (https://www.omicstudio.cn).

For analyses involving multiple comparisons, the Benjamini-Hochberg method was adopted to correct the false discovery rate (FDR), with the significance threshold set at FDR < 0.05. All P-values shown in the multi-omics correlation heatmaps and networks represent FDR-adjusted values. The significance annotation rules were as follows: P < 0.05 (*), P < 0.01 (**), and P < 0.001 (***). Data analysis of the intestinal microbiota was performed using the MegCloud Platform (http://cloud.magigene.com) and Dix-seq (22).

## 3 Results

### 3.1 Effects of emodin on survival rate and clinical symptoms of Yellow River carp infected with *A. hydrophila*

**Figure 1:**
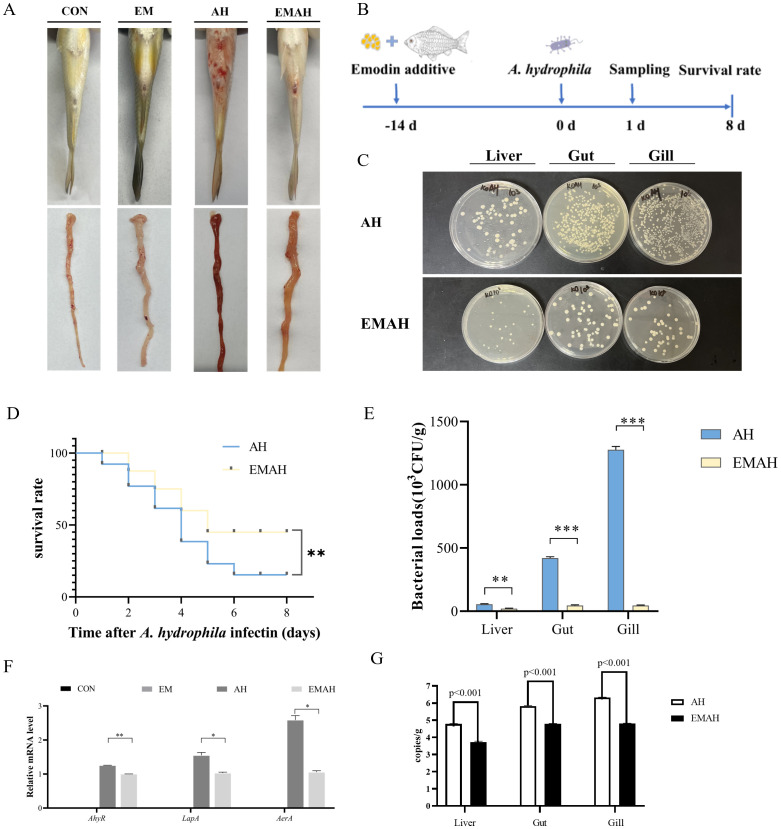
Protective effect of emodin against *Aeromonas hydrophila* infection in Yellow River carp. **(A)** Clinical signs of experimental fish. **(B)** Experiment design. Yellow River carp were orally administered emodin (50 mg/kg/day) via gavage once daily for 14 consecutive days. Following the final administration, the fish were intraperitoneally challenged with *Aeromonas hydrophila* at a concentration of 1 × 10^8^ CFU/mL, administered at a dose of 1 μL per gram of body weight. Mortality was recorded daily over an 8-day period after the bacterial challenge. **(C)** CFU assay plate illustrating the bacterial load of *A. hydrophila* in Yellow River carp from the AH and EMAH treatment groups. In panel C, the plate images were processed solely to remove handwritten non−scientific labels. No bacterial colonies or background were altered. **(D)** Emodin treatment on survival curve of Yellow River Carp infected with *A. hydrophila*. **(E)** Quantitative analysis of *A. hydrophila* load in Yellow River carp. **(F)** Effect of emodin on mRNA expression levels of virulence factors in *A. hydrophila*. **(G)** Comparison of *A. hydrophila* copy numbers in the liver, intestine, and gills between the AH group and the EMAH group. R^2^ = 0.9992. Statistical significance between AH and EMAH groups in panels E, F and G was assessed using an independent samples *t-test* (*P < 0.05, **P < 0.01).

To evaluate the protective effect of emodin against *A. hydrophila* infection, a pathogen challenge test was conducted using Yellow River carp (**Figure 1B**). During the 8-day observation period, no mortality was observed in the CON group, and the fish exhibited normal activity without any discernible clinical symptoms. In the AH group, mortality commenced at 24 h post-infection and increased rapidly between day 2 and day 3, with cumulative mortality reaching 80% by day 8. Infected fish showed typical clinical symptoms including abdominal distension and anal hyperemia. Post-mortem examination revealed pale body color, fin rot, turbid and exophthalmic eyes, hemorrhagic spots on the skin and abdomen, as well as intestinal hyperemia.

Compared with the AH group, fish in the EMAH group exhibited ameliorated clinical symptoms, such as brighter body color, reduced skin hemorrhage, alleviated intestinal and anal hyperemia (**Figure 1A**). Survival analysis showed that the survival rate of the EMAH group was 47.0%, which was significantly higher than that of the AH group (**Figure 1D**). Further analysis revealed significant differences in tissue bacterial load among treatment groups, especially in the intestine, liver and gills (**Figures 1C, E**). The bacterial load in tissues of infected fish was markedly higher than that in the emodin-treated group, with higher loads in the gills and intestine than in the liver. Detection of virulence gene expression of *A. hydrophila* showed that emodin treatment downregulated the mRNA levels of *AhyR*, *LapA* and *AerA* (23) (**Figure 1F**). Further comparative analysis of A. hydrophila copy numbers across different tissues revealed that the bacterial copy numbers in the liver, intestine, and gills of the EMAH group were significantly lower than those in the AH group (**Figure 1G**). These suggest that the protective effect of emodin may be closely associated with these molecular alterations.

### 3.2 Emodin alleviates *A. hydrophila* induced intestinal injury and regulates immune gene expression as well as antioxidant enzyme activities

**Figure 2:**
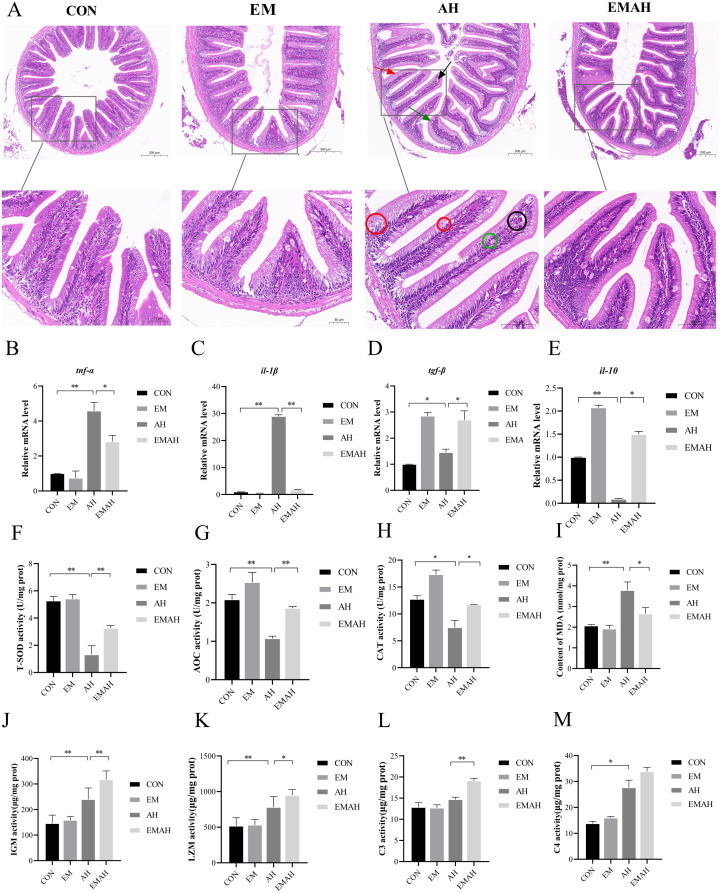
The effect of emodin on gut function of Yellow River carp infected with *A. hydrophila*. **(A)** Intestinal histological structure by H&E (scale bars: 200 and 50 μm), and the black arrow and circles point to hemorrhagic spots, the green arrow and circles indicate goblet cells, the red arrow and circles points to degeneration and necrosis of the intestinal villus epithelium. **(B-E)**. Effect of emodin on gene expression in intestine of Yellow River carp **(B)** *tnf-α*. **(C)** *il-1β*. **(D)** *tgf-β*. **(E)** *il-10*. The Ct values of β-actin across all groups and found no significant differences (P > 0.05) (**Supplementary Figure 8**). Effects of emodin on the gut antioxidant enzyme activities: **(F)** T-SOD activity. **(G)**AOC activity. **(H)** CAT activity. and **(I)** MDA content in Yellow River carp. Effect of emodin on intestinal immunoglobulin in Yellow River carp: **(J)** IgM. **(K)** LZM. **(L)** C3. **(M)** C4. Data are expressed as mean ± SD. Bars with different lowercase letters indicate significant differences among groups (P < 0.05), as determined by one-way ANOVA followed by Duncan’s multiple range test.

Having established that emodin could improve the survival rate, we further investigated its effects on intestinal health. Histopathological analysis showed that the intestinal mucosal epithelium and lamina propria were damaged in the AH group, manifesting as villous epithelial degeneration and necrosis, punctate hemorrhage, decreased number of goblet cells, and fractured intestinal villi. In contrast, such structural injuries were remarkably alleviated in the EMAH group (**Figure 2A**).

In terms of immune-related gene expression, *A. hydrophila* infection upregulated the mRNA levels of genes encoding pro-inflammatory cytokines *tnf-α* and *il-1β*, while downregulating those of the anti-inflammatory cytokine genes *tgf-β* and *il-10* (**Figures 2B–E**). In the EMAH group, the expression levels of pro-inflammatory cytokine genes were lower than those in the AH group, whereas the levels of anti-inflammatory cytokine genes were higher.

Antioxidant system assays revealed that the AH group exhibited increased Malondialdehyde (MDA) content and decreased activities of Superoxide dismutase (SOD), Total antioxidant capacity (T-AOC) and Catalase (CAT). By comparison, the EMAH group showed reduced MDA content and elevated activities of the above antioxidant enzymes (**Figures 2F–I**).

In addition, the activities of immunoglobulin M (IgM), lysozyme (LZM), complement C3 and C4 in the infected group were lower than those in the control group. These indicators were restored to varying degrees in the EMAH group (**Figures 2J–M**).

### 3.3 Emodin alleviates hepatic damage and modulates immune gene expression and antioxidant enzyme activities in Yellow River carp infected with *A. hydrophila*

**Figure 3:**
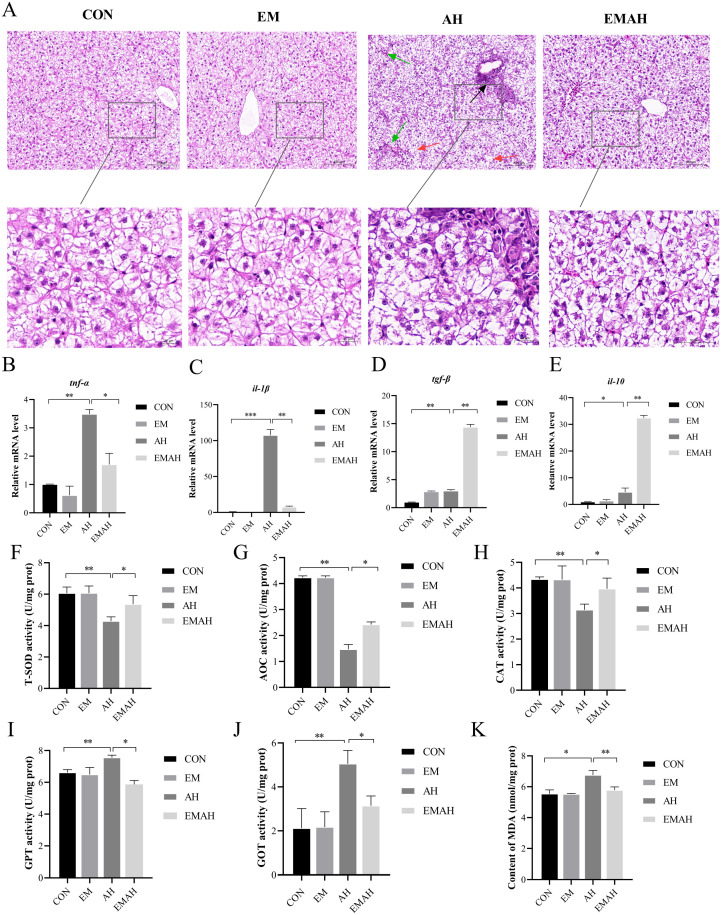
The effect of emodin on liver function of Yellow River carp infected with *A. hydrophila*. **(A)** Liver histopathological analysis of Yellow River carp subjected to emodin and *A. hydrophila* (scale bars: 200 and 50 μm), and the black arrow indicates to inflammatory cell infiltration, the green arrow points to hemorrhagic spots, the red arrow points to cellular edema. **(B-E)**. Effect of emodin on gene expression in the liver of Yellow River carp. **(B)** *tnf-α*. **(C)** *il-1β*. **(D)** *tgf-β*. **(E)** *il-10*. Relative expression levels of the transcript from qRT-PCR were calculated based on the standard curve and normalized to β-actin mRNA level (n = 6). **(F-H)** Effect of oral gavage emodin on the activity of antioxidant enzymes in the liver of Yellow River carp. **(F)** T-SOD activity. **(G)** AOC activity. **(H)** CAT activity. **(I)** GPT activity. **(J)** GOT activity. **(K)** MDA content. Data are expressed as mean ± SD. Significant differences among groups are indicated by different lowercase letters (P < 0.05), as determined by one-way ANOVA followed by Duncan’s multiple range test.

Considering the intricate physiological interactions between the intestine and liver (24), we conducted a further investigation into the hepatoprotective effects of emodin. Hepatic histopathological observations revealed that the AH group exhibited disordered hepatic cord structure, punctate hemorrhage, inflammatory cell infiltration, cellular edema, and pyknosis. These pathological alterations were markedly attenuated in the EMAH group compared with the AH group (**Figure 3A**).

At the molecular level, the mRNA levels of pro-inflammatory cytokines *il-1β* and *tnf-α* were upregulated, while those of anti-inflammatory cytokines *il-10* and *tgf-β* were downregulated in the liver of the AH group. In the EMAH group, the expression of pro-inflammatory cytokines was lower, whereas the levels of anti-inflammatory cytokines were higher than those in the AH group (**Figures 3B–E**).

Antioxidant assays showed that the EMAH group possessed higher hepatic T-AOC, SOD and CAT activities relative to the AH group. Meanwhile, the serum activities of alanine transaminase (GPT/ALT), aspartate transaminase (GOT/AST) as well as MDA content in the AH group were significantly higher than those in the control group, indicating severe hepatocellular injury and oxidative stress. These parameters were reduced in the EMAH group and approached control levels (**Figures 3F–K**). Overall, these results suggest that emodin may alleviate liver damage associated with *A. hydrophila* infection.

### 3.4 Effects of emodin on systemic immune parameters

**Figure 4:**
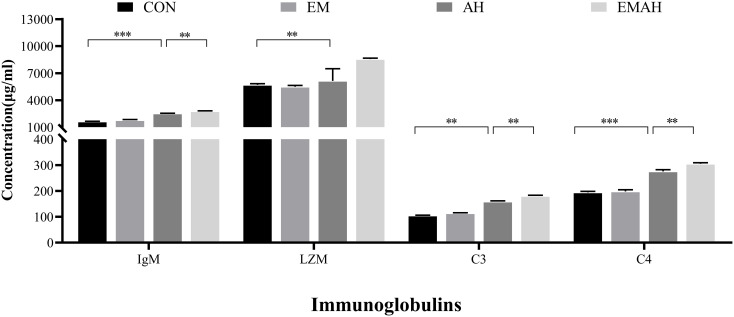
Effects of emodin on blood health of Yellow River carp infected with *A. hydrophila*. The effects of emodin on the serum biochemical indices of Yellow River carp are shown in **Figure 4**.Data are expressed as mean ± SEM (n = 6 biological replicates). Bars with different lowercase letters indicate significant differences among groups (P < 0.05), as determined by one-way ANOVA followed by Duncan’s multiple range test.

Based on the observed local protective effects of emodin on the intestine and liver, this study further explored its influence on systemic immune function. As shown in **Figure 4**, compared with the control group (CON), the activities of serum complement C3, complement C4, IgM and lysozyme (LZM) were significantly elevated in the EMAH group. In addition, the activities of complement C3, C4, and IgM were significantly higher in the EMAH group than in the AH group, whereas LZM activity did not differ significantly between these two groups. These results suggest that emodin may have a certain enhancing effect on the systemic immune-related parameters (particularly the activity of IgM and complement C4) of the Yellow River carp under infection conditions.

### 3.5 Effects of emodin on intestinal microbial community structure of Yellow River Carp infected with *A. hydrophila*

Based on the confirmed effects of emodin on host functions, including local protection of the intestine and liver as well as systemic immune regulation, this study further investigated its impact on the structure of intestinal microbial communities.

#### 3.5.1 Bacterial community structure

**Figure 5:**
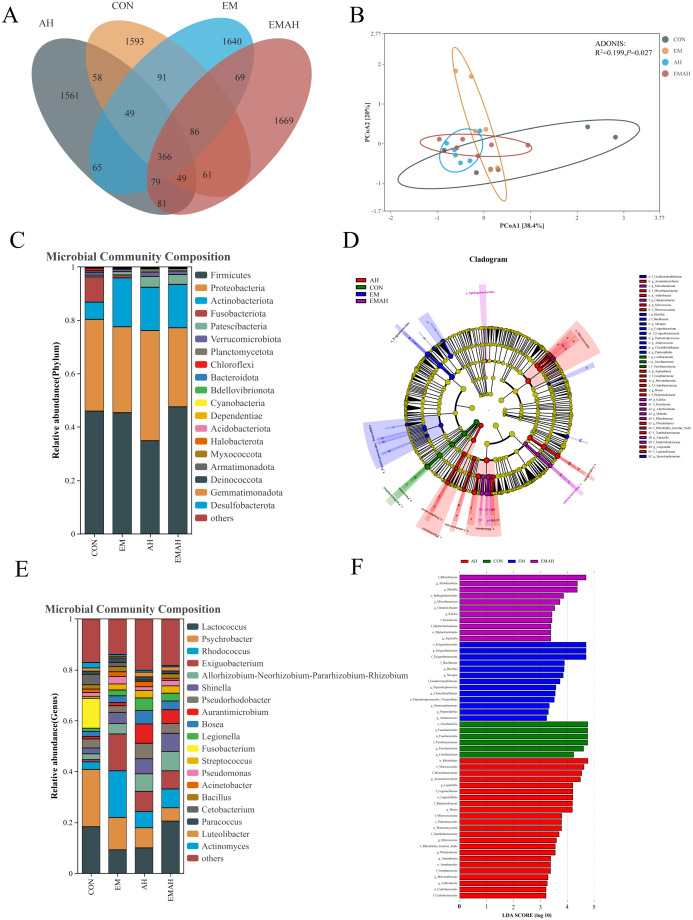
Microbial composition analysis- bacterial community. **(A)** Venn diagram and ASV numbers of intestinal microbiota in the four groups. **(B)** Principal Coordinate Analysis (PCoA) of the microbiota. Each point represents one sample, and the six samples from the same replicate are indicated by the same symbol. **(C)** Composition analysis of intestinal microbiota at the phylum level. **(E)** Composition analysis of intestinal microbiota at the genus level. **(D, F)** Lefse analysis of differential abundant microbial taxa. Here and in the subsequent omics sequencing, we used 6 biological replicates because multiple high-quality journal articles on gut microbiome and differential metabolite studies in similar aquatic animals have adopted 6 biological replicates per group (55, 56). Meanwhile, the Metabolomics Standards Initiative (MSI), an authoritative international guideline in the metabolomics field, clearly recommends a minimum of 3 to 5 biological replicates for biological samples (57).

The ASV analysis identified a total of 7,517 Amplicon Sequence Variants (ASVs) across the four groups. Specifically, the EMAH and AH groups shared 575 ASVs, the CON and AH groups shared 522 ASVs, and the EM and EMAH groups shared 600 ASVs. Notably, the EMAH group exhibited the highest total ASV count (2,460), with 1,669 ASVs unique to this group (**Figure 5A**). The microbiota α-diversity analysis indicated no significant differences among the four groups (**Supplementary Figure 1**). In contrast, the beta-diversity analysis, which assesses the microbial community compositions across groups, demonstrated significant differences among the groups. The proportion of Proteobacteria in the AH group was significantly higher than that in the other groups, as confirmed by the results of the Adonis test (**Figure 5B**). At the phylum level, the five most abundant microbial phyla across the four groups were Firmicutes, Proteobacteria, Actinobacteria, Fusobacteria, and Patescibacteria (**Figure 5C**). The predominant microbial phyla in the CON, EM, AH, and EMAH groups were Firmicutes (46%), Firmicutes (45.45%), Proteobacteria (41.25%), and Firmicutes (47.59%), respectively. Additionally, the proportion of Proteobacteria in the AH group was significantly higher than that in the other groups. At the genus level, the five most prevalent microbial taxa across the four groups were *Lactococcus*, *Psychrobacter*, *Rhodococcus*, *Exiguobacterium*, and *Allorhizobium-Neorhizobium-Pararhizobium-Rhizobium*. The genera exhibiting the highest relative abundance within the CON, EM, AH, and EMAH groups were *Lactococcus* (18.36%), *Rhodococcus* (18.33%), *Lactococcus* (10.04%), and *Lactococcus* (20.56%), respectively (**Figure 5E**). Moreover, the proportion of *Lactococcus* in the EMAH group was significantly elevated compared with the other groups. Significant inter-group differences were also observed in the abundance of genera such as *Bosea* and *Legionella* (**Figures 5D, F**).

#### 3.5.2 Fungi Microbial composition analysis

**Figure 6:**
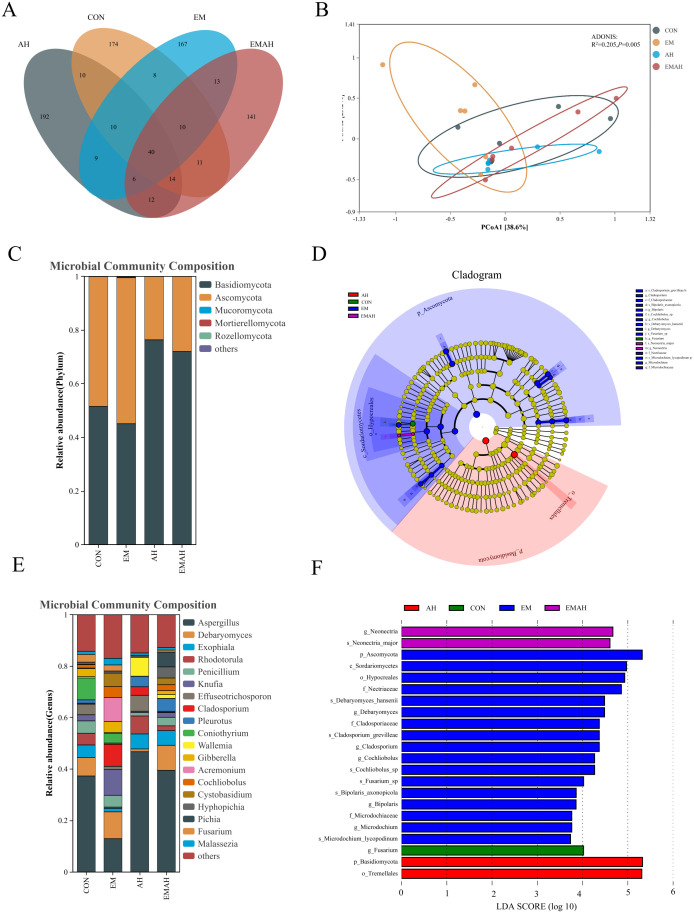
Microbial composition analysis- fungal community. **(A)** Venn diagram and ASV numbers of intestinal microbiota across the four groups. **(B)** The PCoA of the intestinal microbiota composition. Each point represents one sample, and the six samples from the same replicate are indicated by the same symbol. **(C)** Composition analysis of the intestinal microbiota at the phylum level. **(E)** The composition analysis of intestinal microbiota at the genus level. **(D, F)** LEfSe analysis of differentially abundant microbial taxa.

To further investigate the changes in the gut mycobiota, we subsequently analyzed the variations in fungal communities. The ASV analysis revealed a total of 1,435 ASVs across the four groups. Specifically, the EMAH and AH groups shared 72 ASVs, the CON and AH groups shared 74 ASVs, and the EM and EMAH groups shared 69 ASVs. The AH group exhibited the highest ASV count (totaling 293) (**Figure 6A**). In α-diversity analysis, no significant differences were detected among the four groups (**Supplementary Figure 2**). In an analysis of beta diversity, which assesses variations in fungal community composition across different groups, significant differences were identified among the four groups, as indicated by PERMANOVA (**Figure 6B**). At the phylum level, the most abundant fungal phyla were Basidiomycota, Ascomycota, and Mucoromycota (**Figure 6C**). Basidiomycota was the predominant phylum in the CON (51.5%), EM (45.06%), AH (76.34%), and EMAH (72.01%) groups. The presence of *A. hydrophila* infection was associated with an increased abundance of Basidiomycota (76.34%) and a decreased abundance of Ascomycota (23.63%). Furthermore, the additional supplementation of emodin resulted in an increased abundance of Ascomycota (27.98%) and a decreased abundance of Basidiomycota (72.01%) in Yellow River carp. At the fungal genera level, the most abundant fungal taxa across the four groups were *Aspergillus*, *Debaryomyces*, *Exophiala*, *Rhodotorula*, and *Penicillium* (**Figure 6E**). Compared with the CON group, the AH group exhibited an increase in *Aspergillus* abundance (37.24% vs. 46.68%) and a decrease in *Debaryomyces* abundance (7.17% vs. 1.08%). Conversely, a comparative analysis between the AH group and the EMAH group revealed a reduction in the relative abundance of *Aspergillus* (46.68% compared with 39.42%) and an increase in the abundance of *Debaryomyces* (1.08% compared with 9.74%). LEfSe analysis further corroborated that fungal genera such as *Aspergillus* and *Debaryomyces* exhibited significant differences among the groups (**Figures 6D, F**).

### 3.6 Effects of emodin on host metabolomic characteristics after infection

Based on these significant alterations in the intestinal microbiota, we explored the impact of emodin on host metabolism through metabolomic analysis. The quality and reliability of the data were first assessed. In this analysis, a total of 28,746 peaks were retained after data preprocessing. Based on acquired MS² spectra, 3,221 metabolites were finally annotated. The confidence of metabolite identification was graded according to the Metabolomics Standards Initiative (MSI) criteria: Level 1 (matched with authentic standards in MS1, MS2 and RT); Level 2 (matched with public databases in MS1 and MS2); Level 3 (matched with theoretical databases in MS1, MS2 and predicted RT); Level 4 (unknown or MS1−only). In all blank samples, no significant internal standard peaks were detected, indicating good control of material residue and cross−contamination (**Supplementary Figures 3**, **S4**). Pooled quality control (QC) samples showed tight clustering in PCA, indicating good analytical repeatability and instrument stability (**Supplementary Figure 5**).

**Figure 7:**
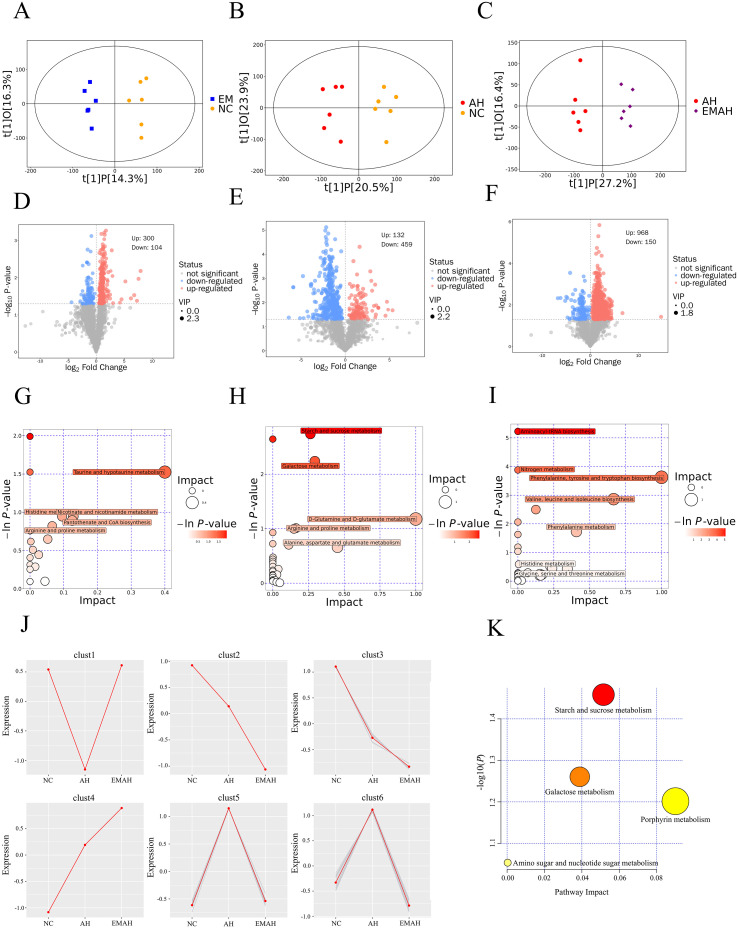
Metabolomics analysis. **(A-C)** Differential metabolites analysis of the intestinal microbiota in Yellow River carp. The significantly altered metabolites were detected with orthogonal projections to latent structures discriminant analysis (OPLS-DA) coefficient plots (variable importance in projection >1.0 and *P <* 0.05). Each symbol represents an individual sample, and samples from the same biological replicate (n=6) are distinguished by identical identifiers. **(D-F)** Volcano plots showing differential metabolites in pairwise comparisons (**(D)** CON vs. EM; **(E)** CON vs. AH; **(F)** AH vs. EMAH). Only MS²-confirmed metabolites (3,221 metabolites) were included. Differential metabolites were defined as those with VIP > 1 and FDR-corrected P < 0.05 (Benjamini-Hochberg method). Red and blue dots represent significantly upregulated and downregulated metabolites, respectively, and gray dots indicate non-significant metabolites. **(G-I)** Pairwise comparison of differential metabolic pathways across the four experimental groups. **(J)** Distribution trends of differential metabolites. **(K)** Pathway enrichment analysis of differential metabolites.

Principal Component Analysis (PCA) demonstrated a lack of overlap in metabolite distribution between the CON and EM groups, the CON and AH groups, and the AH and EMAH groups, indicating distinct variations in the intestinal content metabolomic profiles across the four groups (**Figures 7A–C**). Furthermore, the Partial Least Squares-Discriminant Analysis (PLS-DA) permutation test results indicated that all Q² values were below zero, affirming the robustness of the model (**Supplementary Figure 6**).

The differential metabolite analysis indicated that, relative to the CON group, the EM group exhibited 104 downregulated and 300 upregulated metabolites. In comparison to the AH group, the CON group demonstrated 459 downregulated and 132 upregulated metabolites. Furthermore, when compared with the AH group, the EMAH group showed 150 downregulated and 968 upregulated metabolites (**Figures 7D–F**).

The differential metabolic pathway enrichment analysis revealed that, in the CON versus EM comparison, the differential metabolites were primarily enriched in taurine and hypotaurine metabolism. Taurine, as a prevalent free amino acid, is integral to essential biological processes, including the innate immune response (25, 26). In the CON versus AH comparison, metabolites were enriched in starch and sucrose metabolism, as well as galactose metabolism. In contrast, in the AH versus EMAH comparison, metabolites were predominantly enriched in the pathways of aminoacyl-tRNA biosynthesis, nitrogen metabolism, and phenylalanine, tyrosine, and tryptophan biosynthesis (**Figures 7G–I**), suggesting potential associations with enhanced microbial protein synthesis (27–29).

To better analyze the changing patterns of differential metabolites among the CON, AH, and EMAH groups, a trend analysis was conducted on shared differential metabolites with |FC| ≥ 2 between any two groups, resulting in the identification of six distinct modules (**Figure 7J**). Each module delineates a specific expression trend, which can be broadly categorized into two main patterns: upregulation and downregulation. Differential metabolites from modules 1, 5, and 6 were selected for KEGG enrichment analysis, revealing their predominant involvement in the metabolic pathways of starch and sucrose, porphyrin, and galactose (**Figure 7K**).

### 3.7 Integrated analysis reveals microbiota-metabolism-immunity interactions

**Figure 8:**
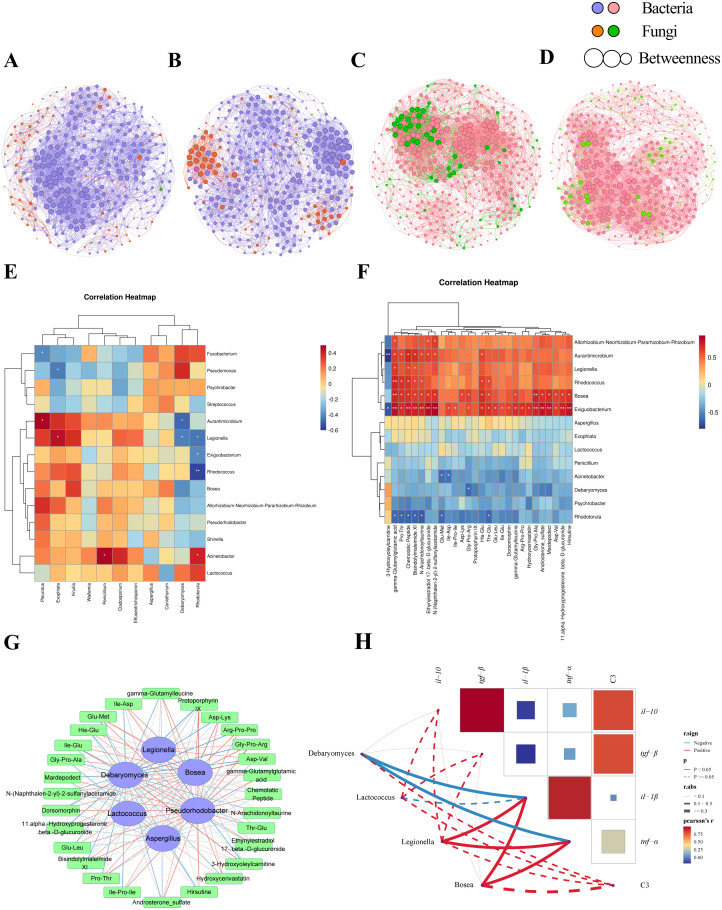
Correlation of central microbiota with immune factors and metabolites Correlation analysis by metabolomics. Fecal microbiota ASV co-occurrence network detected in all test fish analysis in all experimental groups: **(A)** CON. **(B)** EM. **(C)** AH. **(D)** EMAH. Each node corresponds to an ASV. The ASVs from different microbial kingdoms are color-coded to represent their respective groups: bacteria are denoted in pink and blue, while fungi are indicated in orange and green. The size of each node is proportional to its relative abundance and betweenness centrality within the microbial network. Edges denote statistically inferred microbial interactions, which can be either positive or negative. **(E)** Heatmap illustrating species clustering based on bacterial and fungal genus-level abundance. **(F)** Combined analysis of differential flora and metabolites across the four groups. **(G)** Correlation networks between bacterial and fungal genera and metabolites. The greater the number of connecting lines, the closer the species are in their relationships with other species. Microbial-metabolite interactions are represented, with red lines indicating positive correlations blue lines show a negative correlation. **(H)** Correlation networks between central microbiota and immune factors. Red lines represent positive correlations and blue lines represent negative correlations; solid lines represent P < 0.05 and dashed lines represent P > 0.05.

To preliminarily explore the potential relationships among intestinal microbiota, host metabolism and immunity, 16S rRNA sequencing, ITS sequencing, and metabolomic data were integrated for multi-omics correlation analysis in this study. Initially, co-occurrence networks of bacterial and fungal communities across the four groups were constructed using the top 500 ASVs to explore interactions within microbial communities. The number of nodes within these networks was comparable across groups, with 314 nodes in the CON group, 352 in the EM group, 381 in the AH group, and 370 in the EMAH group. Hub ASVs with high or intermediate centrality were identified (**Supplementary Table 2**) to analyze the impact of microbiota shifts on core network nodes. Network complexity, quantified by the connection density between nodes and edges, increased in the AH group after infection but significantly decreased in the EMAH group (**Figures 8A–D**).

Among the CON, AH, and EMAH groups, only one bacterial hub ASV (from *Bosea*, designated B4_Sphingomonas) was identified. The second most prominent ASV in both the EMAH and AH groups was a fungal ASV from the genus *Debaryomyces* (**Supplementary Figure 7**). Comparison of microbial network hubs across the three groups highlighted the core status of bacterial hubs. Analysis of network centrality indices for bacterial and fungal nodes showed that while bacteria dominated the microbial networks in both healthy and experimental groups, fungi also played a significant role in the diseased and prophylactic groups.

To clarify the association between intestinal microbiota and host metabolism, Spearman correlation analysis was performed between genus-level differential microbiota and secondary metabolites. Results showed that the relative abundances of *Debaryomyces* and *Lactococcus* were significantly higher in the EMAH group than in other groups, whereas the abundances of *Bosea* and *Legionella* were lower. Additionally, *Debaryomyces* was positively correlated with *Lactococcus*, and negatively correlated with both *Bosea* and *Legionella* (**Figure 8E**).

Further Spearman correlation analysis revealed that *Debaryomyces* and *Lactococcus* were negatively correlated with several metabolites, including Gly-Pro-Arg, N-oleoyl phenylalanine, gamma-glutamylglutamic acid, 2-hydroxystearate, ricinoleic acid, linarin, and ergothioneine. In contrast, *Bosea* and *Legionella* were positively correlated with these metabolites (**Figures 8F, G**). Furthermore, *Debaryomyces* was negatively correlated with the gene expression of pro-inflammatory cytokines *il-1β* and *tnf-α*, while *Bosea* and *Legionella* were positively correlated with these two pro-inflammatory factors (**Figure 8H**). Collectively, these correlation-based analyses suggest that the protective effects of emodin are associated with significant shifts in specific microbial taxa (including *Debaryomyces*, *Lactococcus*, *Bosea*, and *Legionella*), host metabolic profiles, and immune responses. Whether these microbial and metabolic changes are causal mediators or merely accompanying phenomena requires further investigation.

## 4 Discussion

In intensive aquaculture, green disease prevention and control represents a major bottleneck restricting the sustainable development of the industry. As a unique geographical subspecies in China, the Yellow River carp possesses high economic value and cultural significance, and the conservation of its germplasm resources and the establishment of healthy farming systems have drawn widespread attention. In the present study, a multi-omics integrated analysis strategy was employed to preliminarily investigate the multi-dimensional protective mechanisms through which emodin, a natural product, may help Yellow River carp resist *A. hydrophila* infection. The results showed that emodin was associated with suppressing pathogen virulence expression, enhancing host immune defense and antioxidant barrier functions, and modulating the gut microbiota structure and host metabolic homeostasis. Considering the ability of *A. hydrophila* to survive on fish products and cause illness, the attenuation of its virulence by emodin may also contribute to reducing post-harvest microbial risks.

### 4.1 Association of emodin with pathogen virulence suppression and host basal defense capacity

Yellow River carp pretreated with emodin exhibited improved survival rates following *A. hydrophila* infection, and both hepatic and intestinal tissue damage were alleviated compared with the untreated group. This protective effect may be related to changes in bacterial virulence: in infected fish from the emodin-treated group, the expression levels of the quorum sensing regulatory gene *AhyR* and the virulence genes *LapA* and *AerA* were lower. AhyR is a core transcriptional regulator that affects the expression of virulence factors, and its inhibition may reduce virulence phenotypes such as biofilm formation and protease secretion (30). LapA is critical for bacterial adhesion and tissue invasion (31–33), and AerA is one of the key effector factors for the pathogenicity of *A. hydrophila* (34). Whether emodin directly affects quorum sensing requires further investigation through *in vitro* experiments using sub-inhibitory concentrations, including evaluating bacterial growth, biofilm formation, virulence factor activity, and quorum sensing signals.

Histopathological observations showed that intestinal villus epithelial damage and liver inflammation in infected fish from the emodin-treated group were alleviated compared with the untreated group. At the molecular level, the emodin-treated group exhibited lower expression levels of pro-inflammatory cytokines (*tnf-α*, *il-1β*) and higher expression levels of anti-inflammatory cytokines (*il-10*, *tgf-β*). Meanwhile, this group displayed higher antioxidant activities (T-AOC, SOD, CAT) and liver injury markers (GOT, GPT (35)), suggesting that emodin may be associated with the protection of liver function. The activities of IgM, LZM, and complement components C3 and C4 (36) in the serum were also higher in the emodin-treated group. Together with intestinal antioxidant and morphological parameters, these indicators can reflect, to some extent, the mucosal immune status (37–39).

Previous studies have shown that emodin can reduce mortality in amphioxus (40) and enhance immunity in yellow catfish (41), suggesting that it may exert immunomodulatory effects in aquatic animals. However, related research has mostly focused on its antivirulence effects at low concentrations, and the understanding of its molecular mechanisms remains limited. Building on this foundation, the present study preliminarily explored the mechanisms underlying the protective effects of emodin.

### 4.2 Association of emodin with gut microbiota structure and microecological homeostasis

Gut microbiota plays an important role in host health, involving processes such as nutrient absorption, immune development, and disease susceptibility (3). In this study, we observed the effects of emodin on the gut bacterial and fungal communities of Yellow River carp infected with *A. hydrophila*. Beta diversity analysis showed certain differences in microbial community structure between the emodin-treated group and the control group. At the phylum level, the emodin-treated group exhibited higher relative abundances of Firmicutes (involved in polysaccharide degradation and metabolism) (42), and Ascomycota (associated with improved inflammatory responses and regulation of insulin resistance) (43); meanwhile, the relative abundances of Proteobacteria (associated with dysbiosis and disease occurrence) (44) and Basidiomycota, which increased in abundance after infection, were lower.

At the genus level, the emodin-treated group showed higher relative abundances of *Debaryomyces* (a non-*Saccharomyces* yeast genus with immunomodulatory functions; studies in vertebrates suggest that it can modulate immune cell activity and cytokine secretion patterns (45)) and *Lactococcus* (a lactic acid bacterium that can exert probiotic effects by maintaining an acidic intestinal microenvironment and synthesizing antimicrobial substances (46); whereas the abundances of *Bosea* and *Legionella* (both belonging to Proteobacteria (47)) were lower. Microbial co-occurrence network analysis suggested that the complexity of the gut microbial network was reduced after infection in the emodin-treated group, while the centrality of microbial groups centered on *Debaryomyces* and *Lactococcus* was relatively enhanced within the network.

In the present study, Proteobacteria was the dominant phylum in the gut throughout, while *Aeromonas* did not emerge as a dominant genus, a phenomenon that warrants further discussion. Previous studies have shown that *Aeromonas* could become a dominant genus after 4 days of high-dose intraperitoneal injection (48), and similar results were observed after 4 days of immersion infection (49). In contrast, our study employed a single intraperitoneal injection with a lower dose and sampling at only 24 h post-infection, which may partly explain its low abundance. Moreover, intraperitoneal injection establishes a systemic infection model in which the gut is not the initial colonization site of the pathogen fundamentally different from gut targeted models such as oral gavage or immersion. More importantly, 24 h post-infection represents a critical window for efficient pathogen clearance by the host immune system. It has been reported that inflammatory cytokine pathways are significantly enriched at 24 h after *A. hydrophila* infection (10), and that immune responses are upregulated at 6–18 h but markedly decline by 36 h (50), suggesting that immune clearance is fully activated at 24 h and may have reduced the relative abundance of Aeromonas in the gut. Similar phenomena have been observed in mammalian models; for instance, after intraperitoneal injection of Salmonella in mice, the genus Salmonella also failed to become a dominant gut genus (51). Taken together, we propose that this observation may be attributed to multiple factors, including infection route, dose, sampling time, and host immune clearance, and the exact mechanisms await further experimental verification.

### 4.3 Integrative multi-omics analysis suggests associations between emodin and gut microbiota, metabolism, and immune responses

In this study, we attempted to explore the pathways through which emodin may influence host defense by analyzing the associations between metabolomics and microbiomics. Spearman correlation analysis was used to construct a network among key bacterial genera, metabolites, and immune phenotypes. The results showed that *Debaryomyces* and *Lactococcus*, which were more abundant in the emodin-treated group, were negatively correlated with certain metabolites, whereas *Bosea* and *Legionella* were positively correlated with these metabolites. The abundance of *Debaryomyces* was negatively correlated with the expression levels of the pro-inflammatory cytokines *il-1β* and *tnf-α* in the intestine and liver, while *Bosea* and *Legionella* showed positive correlations. This observation is consistent with some existing findings: *Debaryomyces* possesses immunomodulatory properties and may be associated with the regulation of systemic inflammatory cytokines (45, 52, 53); *in vitro* experiments have also suggested that *Debaryomyces* can affect the production of TNFα and iNOS in Atlantic salmon SHK-1 cells, as well as the secretion of TNFα and IL-10 in primary cultures of head kidney leukocytes (HKL) (45). These results suggest that emodin may be associated with the host inflammatory response.

Further analysis revealed that the bacterial groups that changed more markedly in the emodin-treated group were correlated with various metabolites, including N-arachidonoyl taurine, 3-hydroxyoleoylcarnitine, and glutamate. For example, it has been reported that pathogens can sense glutamate released by the host to enhance their colonization ability (54), suggesting a potential link between emodin-associated microbiota and local intestinal glutamate metabolism, which could hypothetically influence pathogen colonization. This inferred connection is based solely on correlation and remains to be experimentally tested.

This study has certain limitations. All multi-omics analyses were performed at a single time point (24 hours post-infection), reflecting early host-pathogen interactions but failing to capture the temporal dynamics of the responses. Future studies incorporating multiple sampling time points will help provide a more comprehensive understanding of the dynamic processes of microbiota and metabolic changes. Furthermore, the transcript level alone is insufficient to fully reflect immune function, and future validation incorporating the protein level or functional immune cell assays will be more robust. The causal role of key bacterial strains (such as *Debaryomyces*) in the protective effects of emodin can be further explored through germ-free animal models or fecal microbiota transplantation experiments. Importantly, this untargeted metabolomics study is correlative and hypothesis−generating; the identified metabolites require targeted validation with authentic standards in our follow−up studies. Meanwhile, the specific molecular targets and signaling pathways through which specific metabolites from these strains regulate host immune cell function also await in-depth investigation. Overall, this study reveals correlations among gut microbiota, metabolism, and antimicrobial immunity, providing a preliminary basis for subsequent mechanistic studies, but the causal relationships among them remain to be further validated.

## 5 Conclusion

This multi-omics study demonstrates that emodin can effectively enhance the disease resistance of Yellow River carp against *A. hydrophila* infection. The experiment observed that the emodin-treated group exhibited a higher survival rate, milder intestinal and liver damage, and lower expression levels of pathogen virulence genes. Furthermore, the emodin-treated group showed improvements in antioxidant and immune response-related indicators, and the gut microbiota structure and host metabolic profile also differed from those of the untreated group. These findings suggest that emodin could be an environmentally friendly immunostimulant in sustainable aquaculture and might help reduce *A. hydrophila* carriage in farmed fish.

## Data Availability

The datasets presented in this study can be found in online repositories. The names of the repository/repositories and accession number(s) can be found in the article/**Supplementary Material**.
