# Supplementary material for: Emodin treatment is associated with enhanced resistance to Aeromonas hydrophila and correlates with gut microbiota–immune–metabolic modulation in Yellow River Carp: a multi−omics study
Source: Front Immunol. 2026 Jul 15;17:1881196. doi: 10.3389/fimmu.2026.1881196 (PMC13414214; doi:10.3389/fimmu.2026.1881196)
Supplement: Supplementary file 1 [file DataSheet1.docx]

**Figure S1.** **Intestinal bacterial community diversity analysis**

The α diversity analyses of intestinal microbiota among the four groups in the bacterial community. (A) Chao1 index. (B) Shannon index. (C) Simpson index.

**Figure S2.** **Intestinal fungi community diversity analysis**

The α diversity analyses of intestinal microbiota among the four groups in the Fungi community. (A) Chao1 index. (B) Shannon index. (C) Simpson index.

**Figure S3. EIC chromatograms of internal standards in blank samples and QC samples (positive ion mode)**

**Figure S4. EIC chromatograms of internal standards in blank samples and QC samples (negative ion mode)**

**Figure S5. Two-dimensional PCA score plot of QC samples**

**Figure S6.** **Differential analysis of intestinal metabolites between different treatment groups**

Permutation plots of metabolome data processing (A) CON vs EM Group. (B) CON vs AH Group. (C) AH vs EMAH Group.

**Figure S7. Microbial networks of the yellow river carp endosphere microbiotas.**

Fecal microbiota ASV co-occurrence network analysis across all experimental groups: (A) Whole Group. (B) AH Group. (C) EMAH Group. Each node corresponds to an ASV, with edges between nodes indicating positive (blue) or negative (red) correlations, as inferred from the ASV abundance curve using the SparCC method (pseudo*P* < 0.05, correlation values <−0.3 or > 0.3). ASVs from different microbial kingdoms are color codes: bacterial in pink and Fungi in blue. The size of each node reflects its intermediality within the microbiome. Edges between nodes represent predicted interactions.

**Figure S8. The Ct values of β-actin across all groups.**

The Ct values of β-actin in all RT-PCR groups of the intestine (A) and the liver (B)

Figure S1


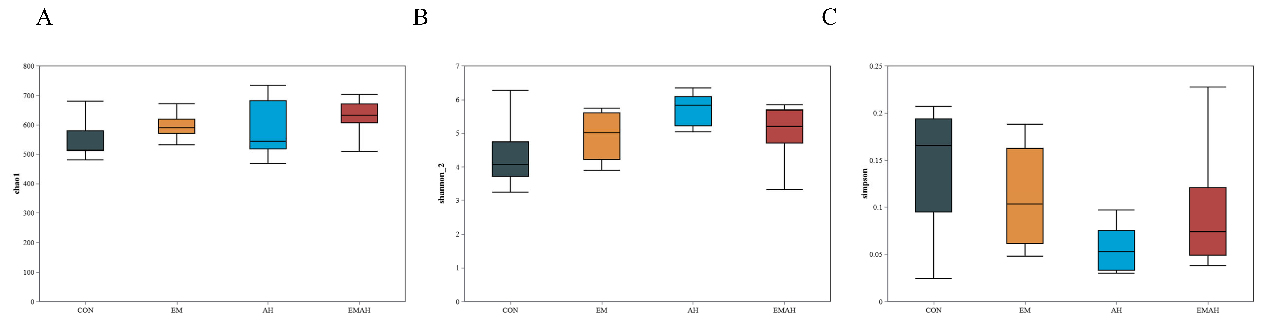


Figure S2


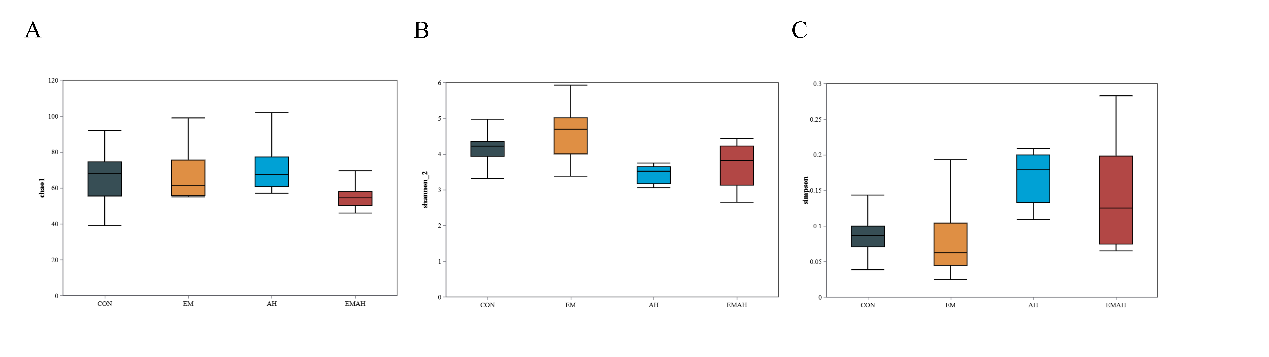


Figure S3


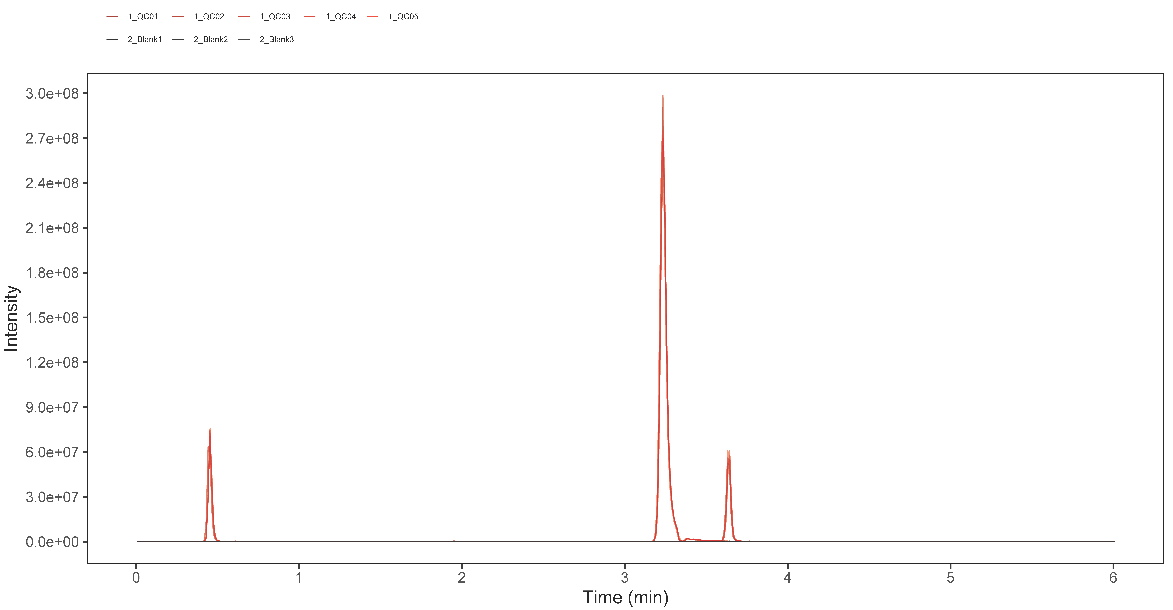


Figure S4


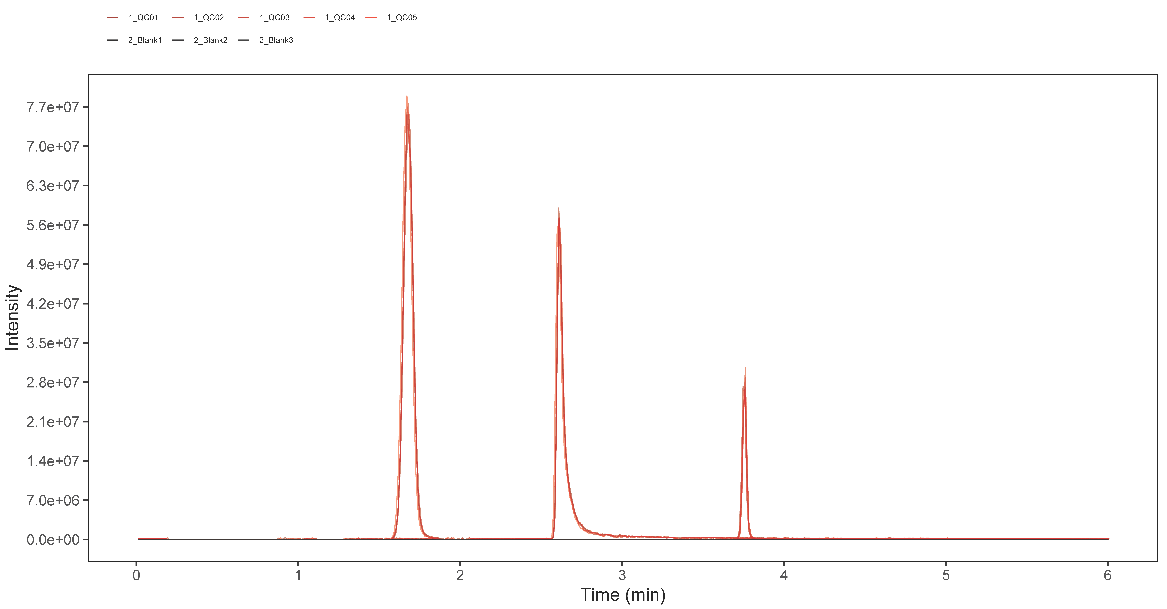


Figure S5


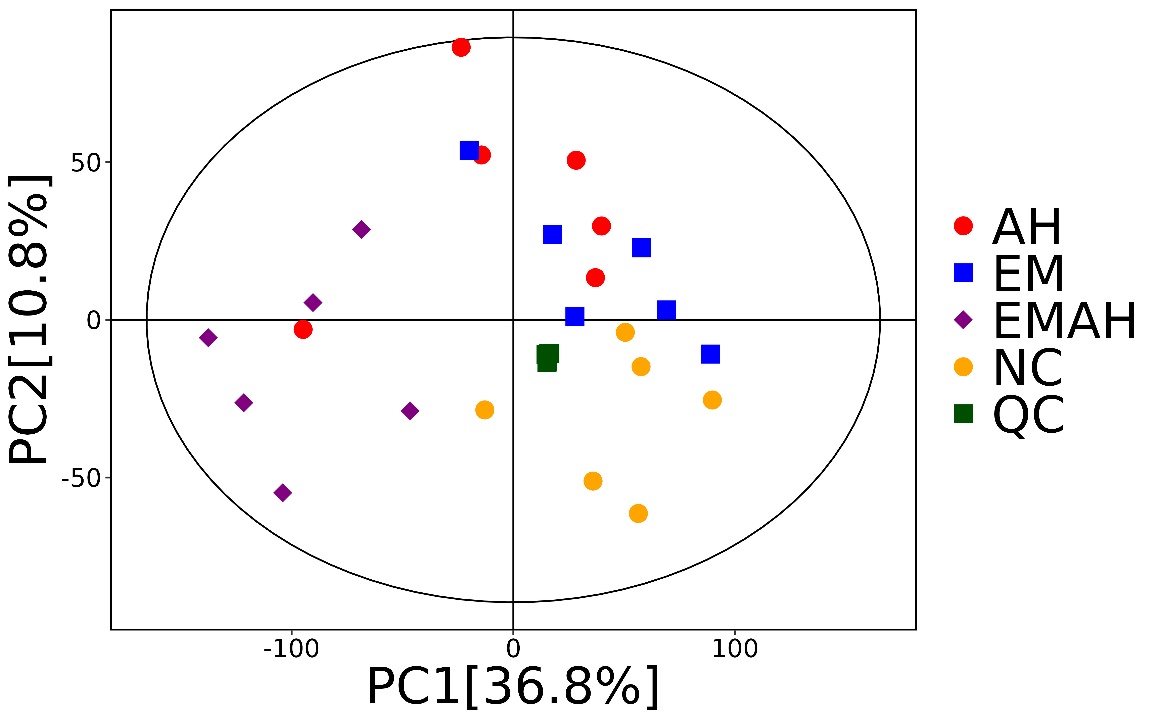


Figure S6


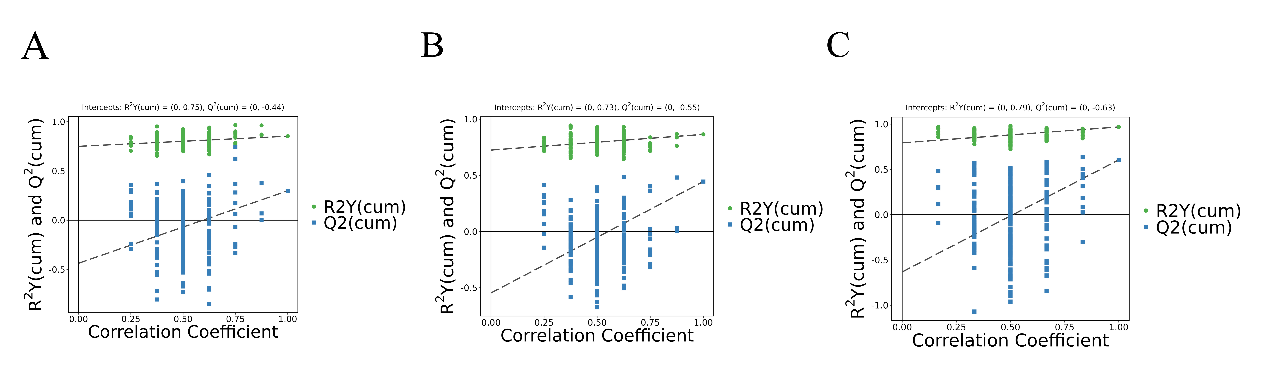


Figure S7


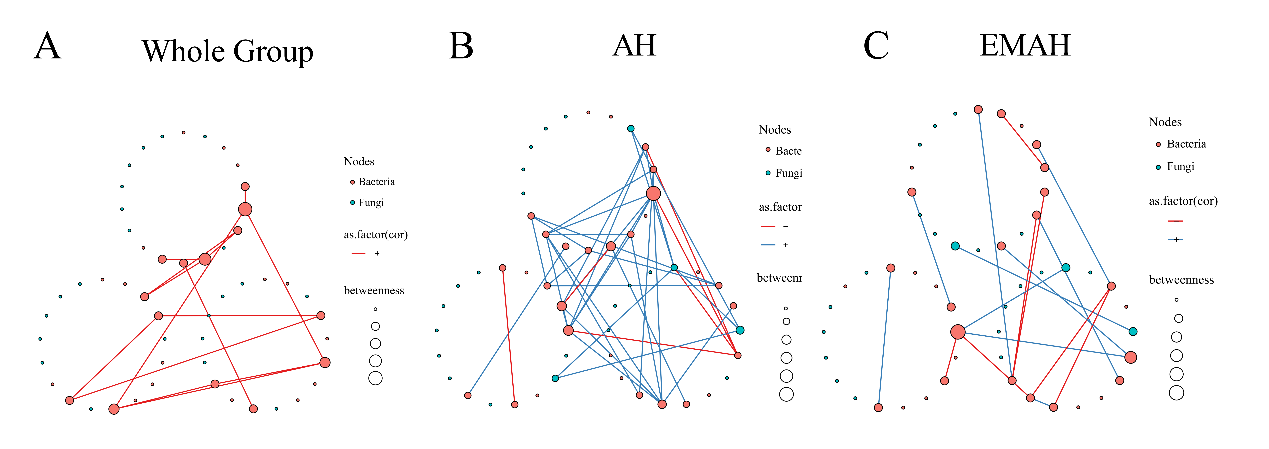


Figure S8


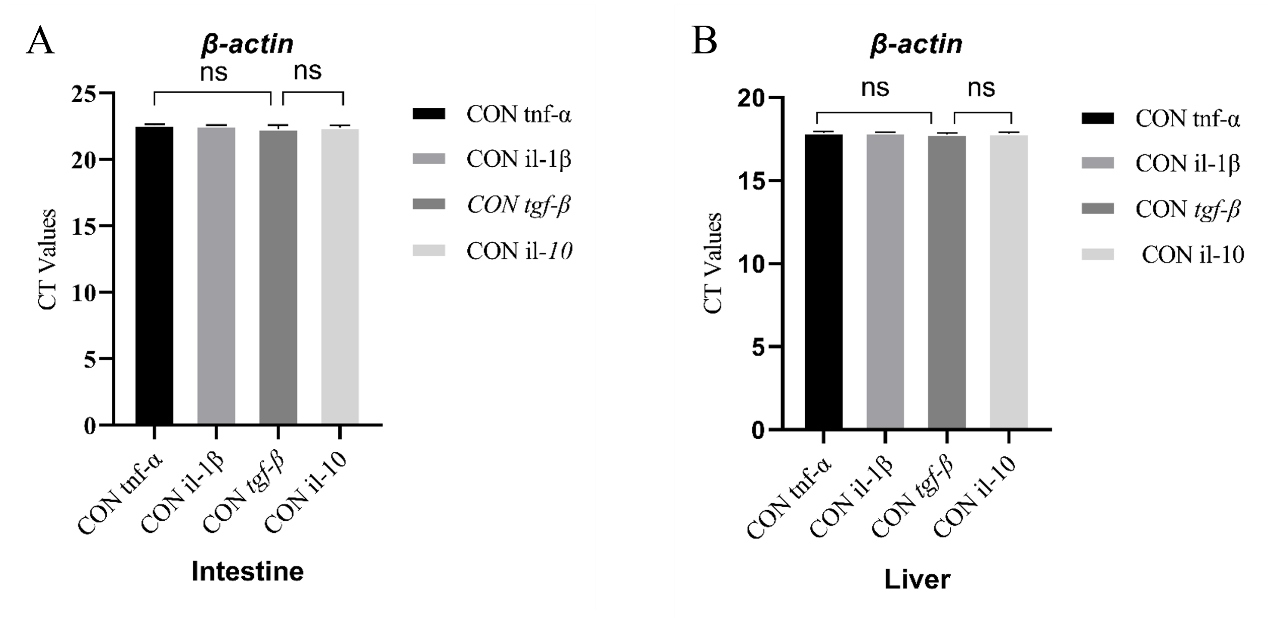


**Table S1. RNA primer sequences**

| **Name** | **Forward primer** **(5′- 3′)** | **Reverse primer** **(5′- 3′)** | **Accession in NCBI** |
| --- | --- | --- | --- |
| *Beta-actin* | CCACCATGTACCCTGGCATT | AGGGCCAGACTCATCGTACT | JQ619774.1 |
| *tnf-α* | CGCGAAGGTGTCAAACAGTG | TTCCGTCACCGGTTTCTACG | AJ311800.2 |
| *il-1β* | AGAAACCGGCACACGTTACA | AAGTTTGTGGTTCGGGTGGT | KC008576.1 |
| *tgf-β* | CCTCACGAGGAGACGAATGC | CTCCTACTGCCCTCTCGTGT | AF056942.1 |
| *il-10* | TGCAAGACTGACTGTTGCTCA | TCTGTTCCACGTTTTCGTCCA | JX524551.1 |
| *AhyR* | TTTACGGGTGACCTGATTGAG | CCTGGATGTCCAACTACATCTT | X89469.1 |
| *LapA* | TTCCACTGGTGCTGGTGTTT | AGAGTGAAAACTCGCCCTGG | ABK39134.1 |
| *AerA* | TCTACCACCACCTCCCTGTC | GACGAAGGTGTGGTTCCAGT | MF198447.1 |

**Table S2. Co-occurrence Network Analysis Data**

| Groups | CON | EM | AH | EMAH |
| --- | --- | --- | --- | --- |
| Nodes | 314 | 352 | 381 | 370 |
| Edges | 3430 | 3097 | 4894 | 4546 |

**Table S3. PCR primer sequences**

| **Name** | **Forward primer** **(5′- 3′)** | **Reverse primer** **(5′- 3′)** | **Accession in NCBI** |
| --- | --- | --- | --- |
| *AerA* | CAAGAACAAGTTCAAGTGGCCA | ACGAAGGTGTGGTTCCAGT | MF198447.1 |
